# Supplementary material for: Effects of exogenous β-glucanase on ileal digesta soluble β-glucan molecular weight, digestive tract characteristics, and performance of coccidiosis vaccinated broiler chickens fed hulless barley-based diets with and without medication
Source: PLoS One. 2021 May 3;16(5):e0236231. doi: 10.1371/journal.pone.0236231 (PMC8092798; doi:10.1371/journal.pone.0236231)
Supplement: Attachment — Submitted filename: OBSERVATIONS PONE-D-20-20511.docx [file pone.0236231.s002.docx]

PONE-D-20-20511

Article Type: Research Article

Full Title: Effects of exogenous β-glucanase on ileal digesta soluble β-glucan molecular weight, digestive tract characteristics, and performance of coccidiosis challenged broiler

chickens fed hulless barley-based diets with and without medication

First of all: the tittle is enormous and I cannot understand what is mileal digesta soluble β-glucan molecular weightw

- Coccidiose vaccine is not the same as coccidiose challenged birds, the dose, the way, many elements are different from a challenge in the field. So I suggest changing the tittle and wherever it is discussed

Abstract:

I cannot understand what is “the soluble β-glucan weight average molecular weight (Mw)” It is impossible to understand what is peak molecular weigh without reading the M&M. Need changing

Line 38- smallest 10% β-glucan molecule (MW-10%) was lower with BGase.

Line 76- I’d like to understand why the decrease in of molecular weight of arabinoxylan is the cause of the decrease and increase of pathogenic/beneficial bacterias

In addition, exogenous xylanase in wheat-based diets increased

the number of gastro-intestinal beneficial bacteria, including lactic acid bacteria, while reducing pathogenic bacteria in broiler chickens [20,21], probably by decreasing the molecular weight of soluble arabinoxylan derived from the wheat.

Introduction- too long, please you need to be more concise

Line 101 to 104 The primary mechanism is generally accepted to be a positive modulation of the diversity and relative abundance of bacteria in the digestive tract microbial community, and thereby the control of enteric disease and stimulation of immune function in broiler chickens [29-31].

I disagree with you. The new theories tell about anti-inflamatory activicty (see Niewold, T. 2007. The nonantibiotic anti-inflammatory effect of antimicrobial growth promoters, the real mode of action? A hypothesis. Poult. Sci. 86:605–609.)

Line 117- ileal digesta soluble β-glucan molecular weight distribution (very confuse)

122	This should result in improved performance of broiler chickens and

Line 135 - The dimensions of the cages were 51 cm in length, 51 cm in width

and 46 cm in height. The grid size of the wire mesh floor of each cage was 2.54 × 2.54 but was covered by a 1.27 × 1.27 cm mesh until d 7. There were two levels of battery cages that were in two rows with back to back cages. The starting 138 room temperature was 32°C, and it was gradually decreased by 2.8°C per week. The minimum light intensity was 25 lux during the experimental period,

Line 141-144 Each cage had a front-mounted feed trough (51 cm in length) and two height-adjustable nipple drinkers. Extra feed and water were supplied to the birds from d 0 to 5 using supplementary chick feeders (50 cm long, plastic) and ice cube trays (16 cells), respectively.

Why all this superfluos details? Is is a paper with few pages. It´s not the entire thesis. I’ve never seen so many specifications.

Experiment 2- how many birds per m square?

Line 153 Additional feed and water were supplied to each pen using a

cardboard egg tray and an ice cube tray, respectively, for the first week. Straw was placed in each room at a thickness of 7.5-10 cm. The room temperature was 33°C at the beginning of the experiment and was gradually reduced to 21°C by d 25. Day length was gradually reduced from 23 h at d 0 to 17 h at d 12, and the light intensity was set to 20 lux at the start of the experiment and gradually decreased to 10 lux by d 10.

No need of such detail

Just say” The feed supply and water were *ad libitum*, and the animals were kept in thermal confort and day length was gradually reduced from 23 h at d 0 to 17 h at d 12.”

Line 163- (Bacitracin- Zoetis Canada Inc.,Kirkland, QC, Canada)

Line 164- t 4.4 mg/kg and Salinomycin Sodium (Phibro Animal Health

165 Corporation, Teaneck, NJ- at 25 mg/kg). two parenthesis do not exist

Line 187 ingestion by the birds. In addition, 60% relative humidity was maintained in the rooms, , to facilitate oocyst cycling.

Line 224- 80°C for β-glucan molecular weight analysis

Line 248- Beta-glucan molecular weight distribution- why the word distribution? Just to confuse the reader

Line 255- weight average molecular weight (Mw) - what is that? Average molecular weight I understand, but weigh average weight no.

Line 258 - Average molecular weight is the average of the molecular weights of all β-glucan

262 by [39] with minor changes. The journal accepts this form of citation?

Line 262 The internal standard for the analysis was made up of 20 ml of 25%. What means internal standard?

Line 299 8.70%, respectively, and the same fractionswere 15.2, 13.7, 1.6 and 0.68%,

Line 306 – 310 Look Table 2 – Mp was not different between 0 Med x 0,1 Med. I don´t see any interaction in the sense the paper is searching. Only for Mp there is an interaction

311 In Experiment 2, interactions were found for all molecular weight criteria at both ages (11 and 33 d) except for Mw at 11 d, which was also unaffected by medication or BGase. No interaction of interes was found for MW-10% ( 33322 a= 26065 a and 7250 b = 1058b) So, no interaction!

**Line 305 Beta-glucan molecular weight**

Only the figures are “distribution”. Avoid this term, except for the figures 1A ,1B, 1C

Line 320 -shown by curve placement relative to the blue line at x-axis point 1e4.

What is point 1e^4^ ?

330 Ileal digesta viscosity was not affected by medication in Experiment 1, but was reduced

331 with the use of BGase. In Experiment 2 at 11 d, an interaction was found between medication and BGase; BGase reduced viscosity without dietary medication. In the interaction, the highest viscosity was noted for the treatment without medication or BGase, and the lowest was the treatments with BGase; treatment with medication and without BGase was intermediate. At d 33 in Experiment 2, BGase decreased viscosity, but there was no medication effect

Medication did not decrease significantly the viscosity (9,73a x 6.04 ab) . It decreased numerically, but not statistically . “a and ab” are not different. This is the interaction that counts

Only the enzyme had an important effect. Please change de sentence

The statistical analysis show interaction because the comparison between 9.73 and 4.13, but this comparison is not important

Line 344- … were also not affected by treatment (Table 5). Noteworthy, the interaction between medication and BGase tended to be significant (*P* = 0.06-0.09) for the concentrations of total and individual SCFA.

Like I said before, you cannot guarantee that the major difference is between 0 Bgase (284) x med (267). Probably the difference appears between 310 and 267 . So reevaluate your analysis to know exactly why there is an interaction. You need a lsmeans analysis to see that

Table 4 jumps to Table 6

Line 348 to 358- the same thing only valeric has an important interaction (table 6)

Line 364 -The molar percentages isobutyric acids were decreased by medication, while enzyme use decreased and increased the proportions of acetic and butyric acids, and valeric acid, respectively. (???)

For Propionic it´s not true: pro – 0 Med ( 49,7b) = Med (36,4 bc)

You cannot use “respectively” comparing two categories and 3 things!!!

Line 366- The interaction between main effects was significant for the proportional isovaleric levels, with enzyme tending to decrease levels in unmedicated diets and increase levels in medicated diets. Although the above effects were significant, differences were small.

Here you used “tending” for a P<0.03 and explains the difference is small??? You are tending your interpretations. No tendency here. This is a significant interaction. In this case medication decreased isov in the absence of the enzyme and the enzyme improved isov only without	medication

Line 430- content weights of the gizzard, jejunum and small intestine. The gizzard content weight to be higher and lower with enzyme use in birds fed non-medicated and medicated diets, respectively.

You are using in wrong sense the word “tendency”. Now-a-days it´s been used for Probabilities between 0.05 and 0.10, not because a mean is similar statistically, although greater or minor numerically (a x ab). In this case, gizzard content without med and enzyme is lower than with med. Only that.

Line 497-… increased gain, while enzyme did not affect gain in birds fed diets without medication and increase gain in the medicated diet. Weight gain from 11 to 22 d was increased by enzyme regardless of diet medication. But med alone also increased WG.

Line 506 Interactions were found between medication and BGase for F:G in all periods.

Medication feed efficiency throughout the trial, but as was the case for BWG, the nature of the interaction with enzyme use changed with bird age. During the 0-11 d period, F:G increased with enzyme use when birds were fed non-medicated diets, but had no effect when the medication was used. For the remainder of the periods, including the total trial, enzyme F:G in birds fed non-medicated diets, but had no effect in broilers consuming medicated diets.

For this response prefer the the words “worse or improve” F:G instead “increase or decrease”, because the nature of the response ( the higher F:G , the worse result)

Line 520 translocation. It can be concluded that the vaccination with Coccivac-B52 induced a disease 521 challenge in the birds from Experiment 2 according to the detailed analysis of mortality data.

Are you not overreacting about this data? The mortality between the two trials was practicaly the same, one with vaccination,the other without vaccination. Besides, ONLY 4,3 % in Exp 2 was due to necrotic enteritis. How do you know that in Exp 1 there was not a cause related to systemic infection, since you did not analyze intestine? For me, you cannot conclude that.

Line 530 With minor exceptions, all three molecular weight responsesfor soluble ileal digesta β-

Parameter has other meaning- A **parameter** is a measurable characteristic of a or a population, such as a mean or a standard deviation …

Line 536 glucan depolymerization is independent of the and the age of the animal. Further,

You cannot say chickens were sick. Vaccination and sickness are completely different things

Line 536- glucan depolymerization is independent of the disease status and the age of the animal. Further, Mw from Experiment 1 also supports the reduction of molecular weight in the ileal digesta soluble β-glucan with the use of BGase.

What is the hypothesis for this sentence? Why Exp 2 would not support the effectiveness of the enzyme? I suggest taking this out

Line 538	In contrast, BGase increased Mw in Experiment 2 in the treatments with antibiotics.

That´s not true. At 11 days, no significant differences

Line 542- The reduction of β-glucan molecular weight and the increased proportion of

small molecular weight soluble β-glucan encourage the assessment of performance and digestive tract characteristics through increased β-glucan fermentation in broilers.

Take out this sentence. No important information is in this sentence.

Line 544 Further, the proportion of small molecular weight β-glucan might be a critical assessment in chickens since chicken ????

Why might be a critical assessment ? For me it is a good response to improve fermentation.

Line 551 utilize fibre [46]. Further, molecular weight parameters were lower in Experiment 1 compared to both ages in Experiment 2.

Since in exp 1, birds were close in age with the second age of Exp 2, how do you interprete this result? Don´t you think medication could be influencing, “protecting” de B glucan of fermenting?

Line 569-… support the difference related to the microbial enzyme activity [48]. To the best of our knowledge, this, , isthe first to document molecular weight. Other researches not published are not valid in this terms.

Line 572 -…BGase effect on the reduction of ileal β-glucan molecular weight in this study is in…

Line 574- The molecular weight responses in the two experiments decreased with medication

Line585 - It demonstrates the higher efficacy of feed BGase in comparison to the BGase originated from microbiota in the chicken gastro-intestinal tract of degrading high molecular weight β-glucan.

What does this sentence mean? I cannot understand

Line 591- ileal viscosity in broiler chickens, you’ve just said that. Don’t need repeat.

Line Nevertheless, viscosity changes at 11 d are similar to the

593 molecular weight data where enzyme decreased values, but the decrease was smaller with medicated diets, and it is primarily due to the low viscosity in the treatments with medication when BGase was not used.

This is wrong. Means are not different between 9.73 a x 6.04 ab

Line 598 - …digesta [56-58]. Further, medication might have shifted the ileal microbial population in a way that leads to increased intestinal mucus production, which can contribute to ileal viscosity [59,60]. In addition, high amounts of NSP in the diet also increase intestinal mucus production in monogastric animals [59,61].

Lot of explanation for no reason. Take out

Line 602 Overall, BGase reduced the empty weights, lengths, and content weights in the digestive tract segments in both experiments, which agrees with previous broiler research that used the same diets but without medication [62].

I don´t agree with you. Some segments were reduced, but not some response so strong that can support this sentence. Modify the sentence , please.

For example, at 11 days (exp 2), SI increased with the enzyme use.

Line 608- … thereby increases digestive function in the broiler chickens [67,68]. Further, HB mediated larger digestive tract might hold more digesta that leads to increased gastro-intestinal content weights in the current study.

Please, show me where there are consistent increase in gastro-intestinal content weights in the current study??

Line 605- … efficiency associated with enzyme use and has been reported previously [63,64]. In addition, the reduction of gastro-intestinal content weights might be associated with increased feed passage rate in the gastro-intestinal tract [65,66]

In your experiment FI was not affected. Increased feed passage rate would increase FI, don´t you think?

In my opinion you are trying to exacerbate same small effects with this long discussion without a data that supports your theory

Line 624 contain BGase since the enzyme also decreases digestive tract size by increasing nutrient digestibility in broiler chickens.

Cannot affirm that, You did not do digestibility of nutrients. Bglucanase did not improve any performance in EXP 1, don’t forget. It actually, tended to DECREASE WG and more, with medication, B glucanase WAS NOT effective in improving BWG, ie, medication has a strong effected than the enzyme

Line 627- BGase and antibiotics on carbohydrate fermentation. Diet BGase and medication depolymerized soluble β-glucan in HB in the ileal digesta of broiler chickens, which may influence carbohydrate fermentation in the lower digestive tract.

You’ve already said that; summarize, please

Line	705- … microbiota [29,92]. Among the beneficial changes is an increase in carbohydrate fermentation [93], including β-glucan, and positively affect gastro-intestinal physiology and health and helps in improving the production performance of broiler chickens. Short chain fatty acids, especially butyric acid, produced as a result of carbohydrate fermentation, increase energy supply to intestinal epithelial cells [94], increases nutrient absorptive surface area by increasing villi size [95,96], and also decreases harmful pathogenic bacteria in the lower digestive tract of chickens [93]. Villi height to crypt depth ratio in the ileum increased with 711 medication in the current study, which supports antibiotics mediated enhancement of the ileal absorptive surface area in broiler chickens. However, total and individual SCFA concentrations in the caeca decreased with the addition of antibiotics, which is contradictory to carbohydrate fermentation induced improvement of physiological and growth parameters in the current research.

Too long this discussion . The intestinal structute results do not show enough news to justify so many discussion

Line 725 - efficiency in the birds aged < 11 d but increased these responses after d 11. Moreover, BGase

Line 735 medication and enzyme on production responses in the battery cage study.
