# Supplementary material for: Effects of exogenous β-glucanase on ileal digesta soluble β-glucan molecular weight, digestive tract characteristics, and performance of coccidiosis vaccinated broiler chickens fed hulless barley-based diets with and without medication
Source: PLoS One. 2021 May 3;16(5):e0236231. doi: 10.1371/journal.pone.0236231 (PMC8092798; doi:10.1371/journal.pone.0236231)
Supplement: Attachment — Submitted filename: Response to Reviewers.docx [file pone.0236231.s003.docx]

We want to express our gratitude to the reviewers and editors for taking the time to volunteer their service to help peer-review this manuscript, and we believe that it will contribute to improving the manuscript.

Editor

| Line | Comment |
| --- | --- |
|  | Please include a copy of Table 10 which you refer to in your text on page 20.  Table 10 was included on page 33 (The table numbers were updated due to the changes in the experiment presentation order). |
|  | We note you have included a table to which you do not refer in the text of your manuscript. Please ensure that you refer to Table 2 in your text  Table 2 was referred to in the text (L297 and L364). |

Reviewer #1

The authors would like to thank the reviewer for taking time from

| Line | Comment |
| --- | --- |
|  | It is meaningful to elucidate the relationship between beta-glucanase and the availability of diets rich in beta-glucan.  However, in this manuscript, I think that only experimental data are only listed overall, and the results are not well organized as research papers. It is necessary to clarify the aim and the background of each experiment and revise the entire manuscript  The results were organized separately under each experiment in line with reviewer comments.  The aim and background of each experiment were described in the introduction (L93-103). |
|  | Terms should not be abbreviated unless necessary. In particular, do not abbreviate general terms consisting of two words.  The following abbreviations were removed from the manuscript; BWG, FI, GC, NSP, TDF, IDF, SDF. |
|  | Please proofread the entire manuscript carefully. The Tables are out of order and duplicates partly.  The entire manuscript was proofread.  The table numbers were updated due to the changes in the presentation of experiments in the entire manuscript. |
| L116-119 | The authors state that the objective of the current study was to investigate the effects under different housing environments and disease conditions, but it seems that the two experiments have not reproduce different housing environments and disease conditions (only vaccinated with a coccidia vaccine).  Birds were housed in cages and on the floor in litter covered pens. These environments alter the nature of the exposure of birds to environmental microbes and the ability of birds to cycle organisms via coprophagy. The cage environment eliminates or greatly reduces coprophagy, and the environment is cleaner because of regular excreta removal. A major difference between the experiments was coccidiosis vaccination, with the degree of response exacerbated by high environmental humidity and litter moisture. The authors conclude that the housing environments and disease exposure are quite different in the two experiments  These differences were clarified in the introduction (L97-103). |
| L126 | What does “environmental pressures” mean?  Deleted. |
| L134 | neonatal chicks? Please clarify the age of birds.  Revised (L122 and L170). |
| L138 | The starting room temperature was … →When the chicks placement, the room temperature was…  The details about environmental conditions, including room temperature, were shorted as requested by Reviewer #2 (L123). |
| L150 | Is it correct that the four treatments are the same as in Experiment 1? The treatment in Experiment 2 should be explained again.  Treatments for the two experiments were described separately in line with the reviewer comments (L127-131 and L 179-183). |
| L179 | Coccidiosis challenge →Coccidia vaccination Vaccines are drugs that prevent the development of coccidiosis. It is not appropriate to interpret the changes caused by vaccination as coccidiosis.  Revised (L191). |
| L191 | Rearing performance?  Revised (L139 and L202). |
|  | Please explain the reason why the composition of the feed used in Experiment 1 and Experiment 2 were different.  Differences in feeds used in the experiments are related to logistical aspects of feed manufacturing and the animals' eventual use.  In Experiment 1, small bird numbers meant that amounts of diet manufactured were small, and it would be challenging to manufacture two pelleted (crumbled) diets. Pelleting diets require more quantity to permit stabilization of pelleting conditions. The authors chose the grower diet, which best represents bird requirements over the entire trial. Experiment 1 birds were euthanized at the end of the trial and did not enter the human food chain.  Because of the much larger numbers of birds in Experiment 2, it was possible to use both starter and grower diets. Birds from this experiment that were not used for tissue collection were marketed to a commercial processing facility.  The Experiment 1 diet (grower) was almost identical to the Experiment 2 grower diet. The authors have not changed the manuscript in response to this reviewer's comment. |
|  | Materials and methods: To understanding easily, materials and methods for Experiment 1 and Experiment 2 should be described separately.  Experiment 1 Birds and Housing Diets Data collection Sample collection  Experiment 2 Birds and Housing Diets Coccidia (not coccidiosis) challenge Data collection Sample collection  Materials and methods for each experiment were separately described as requested by the reviewer. |
|  | The results of Experiment 1 and Experiment 2 should be organized separately.  The results for the two experiments were organized separately under each experiment. |
| L254-255 | Molar mass distribution curves were used to obtain β-glucan (Mp) ,.. ?  The parameters, including Mp, Mw and MW-10%, have been estimated by the software. Revised (L246-248). |
| L262 | ...by Zhao et al. with minor changes.  Revised (L253). |
| L306 -308 | What is the interaction between main effects? Also, what do the value and degree of L307 mean? Please explain using suitable terms.  The description of the interaction was modified for clarity (L295-297). |
| L317 | Fig1 has not mention 33d. Please explain the reason in the materials and methods.  The same distribution patterns were observed for day 33, but only d 11 was presented as an example. This was explained in the results (L374-376). |

Reviewer #2

| Line | Comment |
| --- | --- |
|  | This work is very difficult to read due to the great detail of data (and of a certain repetition), as there are always two types of the same answer (SCFA μmol / g of wet ileal content Molar percentage of total SCFA, Content, Weight, Empty weight ( %) Length (cm / 100g) In addition, the use of expressions that must be corrected such as "soluble β-glucan weight average molecular weight" makes it even more difficult.  The authors have used the correct and standard technical terms to mention the analyzed parameters.  Please note that the presentation order of the sections ‘materials and methods’ and ‘results’ were changed entirely since reviewer #1 has requested to organize Experiment 1 and Experiment 2 separately for these two sections. Therefore, the order of the tables and line numbers were also changed. |
|  | Interpretations about interactions should be reviewed, as there are some with errors.  The interactions were reviewed, and the changes were made when interpreting some of the interactions as mentioned below under reviewer comments (L334-336, L407-408, L501-504). |
|  | I suggest that the introduction and discussion be summarized and use a more objective language to make the article more attractive to the reader. On the other hand, it is data-rich work  The introduction and discussion were shortened and revised (The line numbers range for the respective paragraph is mentioned within brackets).  Introduction;  The first paragraph was shortened (L52-58).  In the second paragraph, the details about fructo-oligosaccharides and mannan-oligosaccharides were removed (L59-73).  The last sentence of the third paragraph was removed (L74-84).  Discussion;  The statement regarding the first time reporting the molecular weight data in the literature was removed (L552-573).  The sentence regarding the gut microbial population in pigs and the details about NSP characteristics and viscosity was removed (L574-587).  The discussion about feed passage time was deleted (L588-605).  The histo-morphology section was shortened (L643-653).  The discussion about butyric acid was eliminated (L667-680). |

PONE-D-20-20511

|  | the tittle is enormous and I cannot understand what is mileal digesta soluble β-glucan molecular weightw  The title was not revised because the authors believe it is clear and descriptive. |
| --- | --- |
|  | Coccidiose vaccine is not the same as coccidiose challenged birds, the dose, the way, many elements are different from a challenge in the field. So I suggest changing the tittle and wherever it is discussed  Changed as coccidiosis vaccination through-out the manuscript, including the title (L 6, 34, 47, 100, 113, 191, 192, 686 and 697). |
|  | Abstract:  I cannot understand what is “the soluble β-glucan weight average molecular weight (Mw)” It is impossible to understand what is peak molecular weigh without reading the M&M. Need changing  Mw was changed as ‘weighted average molecular weight’ in the entire manuscript (L36, 247, 249, and Table 2). The terms ‘weight average’ and ‘weighted average’ molecular weight are used in human nutrition research, and the definition of both terms is the same. The weight/weighted average differs from the number average because the carbohydrate chain’s weight fraction is considered when determining the weighted average. Peak molecular weight was not changed since it is a standard term. The authors believe it is impossible to define them in the abstract because of length, and the definitions were included in the materials and methods with a reference (L249-251). |
| L38 | smallest 10% β-glucan molecule (MW-10%) was lower with BGase.  It was revised as ‘BGase addition’. The definition of MW-10% was not changed, as the authors believe it is clear and descriptive (L39). |
| L76 | I’d like to understand why the decrease in of molecular weight of arabinoxylan is the cause of the decrease and increase of pathogenic/beneficial bacterias  In addition, exogenous xylanase in wheat-based diets increased  the number of gastro-intestinal beneficial bacteria, including lactic acid bacteria, while reducing pathogenic bacteria in broiler chickens [20,21], probably by decreasing the molecular weight of soluble arabinoxylan derived from the wheat.  The reduction of arabinoxylan MW increases beneficial bacteria and is attributed to the increased access of these substrates into the caeca because caecal entry is restricted to small, soluble, and less viscous material, or it can be due to the microbial preference for utilizing small MW carbohydrates over the large MW material. This was not included in the introduction since it will further increase the length. |
|  | Introduction- too long, please you need to be more concise  Introduction was shortened (paragraph line numbers range is included within brackets).  The first paragraph was shortened (L52-58).  In the second paragraph, the details about fructo-oligosaccharides and mannan-oligosaccharides were removed (L59-73).  The last sentence of the third paragraph was removed (L74-84). |
| L101-104 | The primary mechanism is generally accepted to be a positive modulation of the diversity and relative abundance of bacteria in the digestive tract microbial community, and thereby the control of enteric disease and stimulation of immune function in broiler chickens [29-31].  I disagree with you. The new theories tell about anti-inflamatory activicty (see Niewold, T. 2007. The nonantibiotic anti-inflammatory effect of antimicrobial growth promoters, the real mode of action? A hypothesis. Poult. Sci. 86:605–609.)  The theory about the anti-inflammatory activity was also mentioned (L89-90). |
| L117 | ileal digesta soluble β-glucan molecular weight distribution (very confuse)  It is long, but the authors believe it is informative and is necessary to describe the specific parameter measured in the current study. No changes were made. |
| L122 | This should result in improved performance of broiler chickens and  Revised (L109-110). |
| L135 | The dimensions of the cages were 51 cm in length, 51 cm in width  and 46 cm in height. The grid size of the wire mesh floor of each cage was 2.54 × 2.54 but was covered by a 1.27 × 1.27 cm mesh until d 7. There were two levels of battery cages that were in two rows with back to back cages. The starting 138 room temperature was 32°C, and it was gradually decreased by 2.8°C per week. The minimum light intensity was 25 lux during the experimental period,  Line 141-144 Each cage had a front-mounted feed trough (51 cm in length) and two height-adjustable nipple drinkers. Extra feed and water were supplied to the birds from d 0 to 5 using supplementary chick feeders (50 cm long, plastic) and ice cube trays (16 cells), respectively.  Why all this superfluos details? Is is a paper with few pages. It´s not the entire thesis. I’ve never seen so many specifications.  The paragraph was shortened (L121-124). |
|  | Experiment 2- how many birds per m square?  The flock density was added (L171-172). |
| L153 | Additional feed and water were supplied to each pen using a  cardboard egg tray and an ice cube tray, respectively, for the first week. Straw was placed in each room at a thickness of 7.5-10 cm. The room temperature was 33°C at the beginning of the experiment and was gradually reduced to 21°C by d 25. Day length was gradually reduced from 23 h at d 0 to 17 h at d 12, and the light intensity was set to 20 lux at the start of the experiment and gradually decreased to 10 lux by d 10.  No need of such detail  Just say” The feed supply and water were ad libitum, and the animals were kept in thermal confort and day length was gradually reduced from 23 h at d 0 to 17 h at d 12.”  The paragraphs were shortened (L121-124 and L174-177). |
| L163 | (Bacitracin- Zoetis Canada Inc.,Kirkland, QC, Canada)  Revised (L130). |
| L164 | t 4.4 mg/kg and Salinomycin Sodium (Phibro Animal Health  165 Corporation, Teaneck, NJ- at 25 mg/kg). two parenthesis do not exist  Revised (L130-131). |
| L187 | ingestion by the birds. In addition, 60% relative humidity was maintained in the rooms, , to facilitate oocyst cycling.  Revised (L199-200). |
| L224 | 80°C for β-glucan molecular weight analysis  Revised (L165-166). |
| L248 | Beta-glucan molecular weight distribution- why the word distribution? Just to confuse the reader  The word ‘distribution’ was removed through-out the paper. |
| L255 | weight average molecular weight (Mw) - what is that? Average molecular weight I understand, but weigh average weight no.  The weighted average emphasizes the weight fraction of the molecule/carbohydrate chain. A weighted average is more accurate than a number average because all numbers in a data set are assigned an identical weight. The definition was included in the materials and methods (L249-251).  Number average molecular weight = ∑ N_i_M_i_/ ∑ N_i_  Weight or weighted average molecular weight = ∑ N_i_M_i_^2^/ ∑ N_i_M_i_  M_i_ is the weight of the molecule i  N_i_ is the number of molecules of weight M |
| L258 | Average molecular weight is the average of the molecular weights of all β-glucan  Revised (L250). |
| L262 | ...by Zhao et al. with minor changes.  Revised (L253). |
| L262 | The internal standard for the analysis was made up of 20 ml of 25%. What means internal standard?  The internal standard is a known concentration of a substance present in every sample that is analyzed. Internal standards are commonly used in chromatography. |
| L299 | 8.70%, respectively, and the same fractionswere 15.2, 13.7, 1.6 and 0.68%,  Revised (L291). |
| L306-310 | Look Table 2 – Mp was not different between 0 Med x 0,1 Med. I don´t see any interaction in the sense the paper is searching. Only for Mp there is an interaction  Interactions are found for Mp in both experiments (L295-297 and L363-367). Please check the P values and mean separation letters in Table 2. No changes were made. |
| L311 | In Experiment 2, interactions were found for all molecular weight criteria at both ages (11 and 33 d) except for Mw at 11 d, which was also unaffected by medication or BGase. No interaction of interes was found for MW-10% ( 33322 a= 26065 a and 7250 b = 1058b) So, no interaction!  Interactions are found for MW-10% at both ages (L 366-368). Please see the P values and mean separation in Table 2. No changes were made. |
| L305 | Beta-glucan molecular weight  Only the figures are “distribution”. Avoid this term, except for the figures 1A ,1B, 1C  The word ‘distribution’ was removed. |
| L320 | shown by curve placement relative to the blue line at x-axis point 1e4.  What is point 1e4 ?  1e^4^ is a random point that is selected to compare the three graphs since the X-axis is different in the graphs. It was mentioned in the text (L372-373). |
| L330 | Ileal digesta viscosity was not affected by medication in Experiment 1, but was reduced  Revised (L303). |
| L331 | with the use of BGase. In Experiment 2 at 11 d, an interaction was found between medication and BGase; BGase reduced viscosity without dietary medication. In the interaction, the highest viscosity was noted for the treatment without medication or BGase, and the lowest was the treatments with BGase; treatment with medication and without BGase was intermediate. At d 33 in Experiment 2, BGase decreased viscosity, but there was no medication effect  Medication did not decrease significantly the viscosity (9,73a x 6.04 ab) . It decreased numerically, but not statistically . “a and ab” are not different. This is the interaction that counts  Only the enzyme had an important effect. Please change de sentence  The authors agree that ‘a’ and ‘ab’ are not statistically significant, which is why it was stated as ‘BGase reduced viscosity without dietary medication’ (L382-383). The authors have not mentioned ‘medication decreasing the viscosity at d 11’. No changes were made. |
| L344 | … were also not affected by treatment (Table 5). Noteworthy, the interaction between medication and BGase tended to be significant (P = 0.06-0.09) for the concentrations of total and individual SCFA.  Like I said before, you cannot guarantee that the major difference is between 0 Bgase (284) x med (267). Probably the difference appears between 310 and 267 . So reevaluate your analysis to know exactly why there is an interaction. You need a lsmeans analysis to see that  The authors have just mentioned that interaction tended to be significant based on the P values and did not mention the difference between specific means (L313-314). No changes were made. |
|  | Table 4 jumps to Table 6  All the table numbers were updated since the presentation of results changed in the entire manuscript. |
| L348-358 | the same thing only valeric has an important interaction (table 6)  The authors have mentioned the interaction is found only for valeric acid, and the other mentioned interactions trended to be significant. No changes were made. |
| L364 | The molar percentages isobutyric acids were decreased by medication, while enzyme use decreased and increased the proportions of acetic and butyric acids, and valeric acid, respectively. (???)  For Propionic it´s not true: pro – 0 Med ( 49,7b) = Med (36,4 bc)  You cannot use “respectively” comparing two categories and 3 things!!!  Revised (L403-405). |
| L366 | The interaction between main effects was significant for the proportional isovaleric levels, with enzyme tending to decrease levels in unmedicated diets and increase levels in medicated diets. Although the above effects were significant, differences were small.  Here you used “tending” for a P<0.03 and explains the difference is small??? You are tending your interpretations. No tendency here. This is a significant interaction. In this case medication decreased isov in the absence of the enzyme and the enzyme improved isov only without medication  The authors agree that it is a significant interaction, and the changes were made in the text (L407-408). |
| L430 | content weights of the gizzard, jejunum and small intestine. The gizzard content weight to be higher and lower with enzyme use in birds fed non-medicated and medicated diets, respectively.  You are using in wrong sense the word “tendency”. Now-a-days it´s been used for Probabilities between 0.05 and 0.10, not because a mean is similar statistically, although greater or minor numerically (a x ab). In this case, gizzard content without med and enzyme is lower than with med. Only that.  The authors agree with the argument, and the changes were made in the text (L334-336). |
| L497 | … increased gain, while enzyme did not affect gain in birds fed diets without medication and increase gain in the medicated diet. Weight gain from 11 to 22 d was increased by enzyme regardless of diet medication. But med alone also increased WG.  Revised (L501-504). |
| L506 | Interactions were found between medication and BGase for F:G in all periods.  Medication feed efficiency throughout the trial, but as was the case for BWG, the nature of the interaction with enzyme use changed with bird age. During the 0-11 d period, F:G increased with enzyme use when birds were fed non-medicated diets, but had no effect when the medication was used. For the remainder of the periods, including the total trial, enzyme F:G in birds fed non-medicated diets, but had no effect in broilers consuming medicated diets.  For this response prefer the the words “worse or improve” F:G instead “increase or decrease”, because the nature of the response ( the higher F:G , the worse result)  Changed (L514-518). |
| L520 | translocation. It can be concluded that the vaccination with Coccivac-B52 induced a disease 521 challenge in the birds from Experiment 2 according to the detailed analysis of mortality data.  Are you not overreacting about this data? The mortality between the two trials was practicaly the same, one with vaccination,the other without vaccination. Besides, ONLY 4,3 % in Exp 2 was due to necrotic enteritis. How do you know that in Exp 1 there was not a cause related to systemic infection, since you did not analyze intestine? For me, you cannot conclude that.  The authors agree with the points made by the reviewer and removed the statement about the disease challenge. |
| L530 | With minor exceptions, all three molecular weight responsesfor soluble ileal digesta β-  Parameter has other meaning- A parameter is a measurable characteristic of a or a population, such as a mean or a standard deviation …  Changed it as ‘molecular weight responses’ (L531). |
| L536 | glucan depolymerization is independent of the and the age of the animal. Further,  You cannot say chickens were sick. Vaccination and sickness are completely different things  The ‘disease status and age’ was changed as ‘vaccination status’ (L537). |
| L536 | glucan depolymerization is independent of the disease status and the age of the animal. Further, Mw from Experiment 1 also supports the reduction of molecular weight in the ileal digesta soluble β-glucan with the use of BGase.  What is the hypothesis for this sentence? Why Exp 2 would not support the effectiveness of the enzyme? I suggest taking this out  In Experiment 2, Mw increased with the enzyme at d 33 (and numerically at d 11) in the medicated treatments. It was mentioned in the next sentences, and a sentence was added, mentioning the possible reasons (L543-545). |
| L538 | In contrast, BGase increased Mw in Experiment 2 in the treatments with antibiotics.  That´s not true. At 11 days, no significant differences  It was changed as ‘at d 33’ instead of ‘both days’ (L539-540). |
| L542 | The reduction of β-glucan molecular weight and the increased proportion of  small molecular weight soluble β-glucan encourage the assessment of performance and digestive tract characteristics through increased β-glucan fermentation in broilers.  Take out this sentence. No important information is in this sentence.  The authors are of the opinion that the sentence connects molecular weight results with other responses in the research. Therefore, the sentence was not removed, but it was revised to clarify the concept (L545-548) hopefully. |
| L544 | Further, the proportion of small molecular weight β-glucan might be a critical assessment in chickens since chicken ????  Why might be a critical assessment ? For me it is a good response to improve fermentation.  Revised (L548-549). |
| L551 | utilize fibre [46]. Further, molecular weight parameters were lower in Experiment 1 compared to both ages in Experiment 2.  Since in exp 1, birds were close in age with the second age of Exp 2, how do you interprete this result? Don´t you think medication could be influencing, “protecting” de B glucan of fermenting?  The same medication has been used in both experiments and could not affect molecular weight differences in the two experiments. The authors have mentioned the possible reasons in the text (L560-573). No changes were made. |
| L569 | … support the difference related to the microbial enzyme activity [48]. To the best of our knowledge, this, , isthe first to document molecular weight. Other researches not published are not valid in this terms.  The sentence was deleted. |
| L572 | …BGase effect on the reduction of ileal β-glucan molecular weight in this study is in…  Revised (L572). |
| L574 | The molecular weight responses in the two experiments decreased with medication  Revised (L574). |
| L585 | It demonstrates the higher efficacy of feed BGase in comparison to the BGase originated from microbiota in the chicken gastro-intestinal tract of degrading high molecular weight β-glucan.  What does this sentence mean? I cannot understand  The sentence was removed. |
| L591 | ileal viscosity in broiler chickens, you’ve just said that. Don’t need repeat.  Revised (L584-585). |
| L593 | Nevertheless, viscosity changes at 11 d are similar to the molecular weight data where enzyme decreased values, but the decrease was smaller with medicated diets, and it is primarily due to the low viscosity in the treatments with medication when BGase was not used.  This is wrong. Means are not different between 9.73 a x 6.04 ab  The sentence was deleted. |
| L598 | …digesta [56-58]. Further, medication might have shifted the ileal microbial population in a way that leads to increased intestinal mucus production, which can contribute to ileal viscosity [59,60]. In addition, high amounts of NSP in the diet also increase intestinal mucus production in monogastric animals [59,61].  Lot of explanation for no reason. Take out  Deleted. |
| L602 | Overall, BGase reduced the empty weights, lengths, and content weights in the digestive tract segments in both experiments, which agrees with previous broiler research that used the same diets but without medication [62].  I don´t agree with you. Some segments were reduced, but not some response so strong that can support this sentence. Modify the sentence , please.  For example, at 11 days (exp 2), SI increased with the enzyme use.  Revised (L588). |
| L608 | … thereby increases digestive function in the broiler chickens [67,68]. Further, HB mediated larger digestive tract might hold more digesta that leads to increased gastro-intestinal content weights in the current study.  Please, show me where there are consistent increase in gastro-intestinal content weights in the current study??  The sentence was deleted. |
| L605 | … efficiency associated with enzyme use and has been reported previously [63,64]. In addition, the reduction of gastro-intestinal content weights might be associated with increased feed passage rate in the gastro-intestinal tract [65,66]  In your experiment FI was not affected. Increased feed passage rate would increase FI, don´t you think?  In my opinion you are trying to exacerbate same small effects with this long discussion without a data that supports your theory  The sentence was deleted. |
| L624 | contain BGase since the enzyme also decreases digestive tract size by increasing nutrient digestibility in broiler chickens.  Cannot affirm that, You did not do digestibility of nutrients. Bglucanase did not improve any performance in EXP 1, don’t forget. It actually, tended to DECREASE WG and more, with medication, B glucanase WAS NOT effective in improving BWG, ie, medication has a strong effected than the enzyme  Removed this part of the sentence - ‘by increasing nutrient digestibility in broiler chickens’. |
| L627 | BGase and antibiotics on carbohydrate fermentation. Diet BGase and medication depolymerized soluble β-glucan in HB in the ileal digesta of broiler chickens, which may influence carbohydrate fermentation in the lower digestive tract.  You’ve already said that; summarize, please  Revised (L606-608). |
| L705 | Too long this discussion . The intestinal structute results do not show enough news to justify so many discussion  This part of the discussion was shortened (L643-653). |
| L725 | efficiency in the birds aged < 11 d but increased these responses after d 11. Moreover, BGase  Revised (L681-682). |
| L735 | medication and enzyme on production responses in the battery cage study.  Revised (L698-699). |
