# Supplementary material for: Effects of exogenous β-glucanase on ileal digesta soluble β-glucan molecular weight, digestive tract characteristics, and performance of coccidiosis vaccinated broiler chickens fed hulless barley-based diets with and without medication
Source: PLoS One. 2021 May 3;16(5):e0236231. doi: 10.1371/journal.pone.0236231 (PMC8092798; doi:10.1371/journal.pone.0236231)
Supplement: Attachment — Submitted filename: PONE revision 2.docx [file pone.0236231.s004.docx]

PONE-D-20-20511

Article Type: Research Article

Full Title: Effects of exogenous β-glucanase on ileal digesta soluble β-glucan molecular weight, digestive tract characteristics, and performance of coccidiosis challenged broiler

chickens fed hulless barley-based diets with and without medication

Revision 1

152 … Total ileal and caecal contents were collected into plastic centrifuge tubes and stored at -20°C for the analysis of SCFA.

160 … Total ileal contents were collected into plastic snap-cap vials (pooled from all the 161 birds in a cage) and centrifuged for 5 min at 17013 × g using a Beckman microfuge (Model E 162 348720, Beckman Instruments, INC, Palo Alto, CA).

How it is possible? Two **total ileal contents** collected in different places? Different birds?

149 …. Two birds were used

153 …. Two

Birds were used to collect ...

The same two birds? If are the same do not repete. If they are different, say it.

364 for Mw at 11 d, which was also unaffected by medication or BGase (Table 2).

Why also?
