# Supplementary material for: Effects of exogenous β-glucanase on ileal digesta soluble β-glucan molecular weight, digestive tract characteristics, and performance of coccidiosis vaccinated broiler chickens fed hulless barley-based diets with and without medication
Source: PLoS One. 2021 May 3;16(5):e0236231. doi: 10.1371/journal.pone.0236231 (PMC8092798; doi:10.1371/journal.pone.0236231)
Supplement: Attachment — Submitted filename: Response to Reviewers.docx [file pone.0236231.s005.docx]

The authors would like to thank the editor and reviewers for taking time to review the manuscript and for the comments.

Reviewer #1

| Line | Comment |
| --- | --- |
|  | Thank you for correcting the overall structure of the manuscript and the points pointed out. This is a treatise with a large amount of data submitted. In the abstract, it is recommended that you briefly summarize the points in the order of Experiment 1, Experiment 2, and Conclusion to make the content easier for the reader to understand.  The abstract was organized in the order of Experiment, 1, Experiment 2 and then conclusion (L 33-45). |
| L 30 | Broilers were… →In the present study, broilers were…  Revised (L 30). |
| L 32 | The reviewer recommends correcting the below sentences as follows. "In Experiment 1, 160 broilers were assigned to 10 cages from day 0 to day 28; we investigated the effects of BGase treatment and the effects of medication. The soluble β-glucan weighted average molecular weight (Mw) in the ileal digesta was … for the smallest 10% β-glucan (MW-10%) was lower with BGase addition."  The sentences were revised (L 33-34). |
| L 33-35 | The reviewer recommends the sentences of L33-L35(“In Experiment 2, broilers (2376) were housed in…in each of nine rooms.”) are moved to the next location of the Experiment 1.  The sentences were moved (L 37-39). |
| L 98-103 | The sentences of “Experiment 1 was completed in … and microbial exposure.” explain to perform in the current study. The reviewer recommends the sentences move to the next location of L104-107.  The sentences were moved as requested (L 103-108). |
| L 414, 421, 500 | What is "main effect"? The authors should need to present them specifically. or, for example, "No effect of the interactions of BGase and medications were found for …"  Main effects were clarified in the sentences (L 337, 405, 420, 429, 437, 487, 520). |

Reviewer #2

| Line | Comment |
| --- | --- |
|  | The article improved with the corrections made and it is interesting as I already said, although very large. However, I do not understand that it should be published in this way, as there is a wrong interpretation of the meaning of the interactions. In statistics, when there are interactions, it is necessary to know how to interpret them. The fact that ANOVA shows that there is an interaction between two or more factors is not enough for the interaction to be discussed in order to prove the hypothesis of a work, since the means test must also show the same. The authors are studying the interaction between enzyme and medication. The interaction that counts is whether the enzyme acts in way A with the drug or acts in way B without the drug. This never happened (and the averaging test clearly shows). I'll give you an example: in table 2, Mp (Exp 1) was higher with medication and lower with enzymes. Enzymes + Medication did not change this fact, since the Mp is numerically higher (10401) than without medication (7793), both, by means test are equal (same letter). Therefore, it is not possible to state that using enzymes alone, decreases Mp more than using an enzyme with medication. One solution would be to use a less demanding means test, in which case increasing the chance of a Type I error. However, with the current statistics, my vote is for rejection  The authors reviewed all the interaction interpretations in the results section (tables and text) and the necessary changes were done in the wording to clarify the interactions according to mean separation (L 297-303, 334-335, 341-342, 372-376, 393-395, 409-412, 422-423, 469-471, 488-489, 531-532). |

PONE-D-20-20511

| Line | Comment |
| --- | --- |
| L 152, 160-161 | … Total ileal and caecal contents were collected into plastic centrifuge tubes and stored at -20°C for the analysis of SCFA.  … Total ileal contents were collected into plastic snap-cap vials (pooled from all the birds in a cage) and centrifuged for 5 min at 17013 × g using a Beckman microfuge (Model E 162 348720, Beckman Instruments, INC, Palo Alto, CA).  How it is possible? Two total ileal contents collected in different places? Different birds?  There were 4 birds in each cage. Two birds per cage was used for pH and SCFA sample collections. Total ileal content from each bird was collected to a tray and only a portion of it was added to the centrifuge tube after mixing. The rest of the ileal content from these two birds was put into the plastic snap cap vial for centrifugation. Another two birds per cage were used for digestive tract morphology data, and the ileal contents from these two birds were also added to the same snap-cap vial. So, ileal contents from all 4 birds per cage were pooled before centrifugation and used for viscosity and molecular weight analyses. The sentences were revised for a clearer explanation (L 154-156, 162-163). |
| L 149, 153 | …. Two birds were used  …. Two  Birds were used to collect ...  The same two birds? If are the same do not repeat. If they are different, say it.  There were 4 birds in each cage. Two birds were used for pH and SCFA sample collection. Another two birds were used for digestive tract morphology data collection. They are different. The sentence was revised (L 156). |
| L 364 | for Mw at 11 d, which was also unaffected by medication or BGase (Table 2).  Why also?  Deleted the word ‘also’ (L 371). |
