# Supplementary material for: Effects of exogenous β-glucanase on ileal digesta soluble β-glucan molecular weight, digestive tract characteristics, and performance of coccidiosis vaccinated broiler chickens fed hulless barley-based diets with and without medication
Source: PLoS One. 2021 May 3;16(5):e0236231. doi: 10.1371/journal.pone.0236231 (PMC8092798; doi:10.1371/journal.pone.0236231)
Supplement: Attachment — Submitted filename: Response to Reviewers.docx [file pone.0236231.s006.docx]

The authors would like to thank the editor and reviewer for taking time to review the manuscript and for the comments.

**Journal requirements:**

The following changes were made to the reference list.

- DOI was inserted/revised in the following references.

50.	Donaldson GP, Lee Sm, Mazmanian SK. Gut biogeography of the bacterial microbiota. Nat Rev Microbiol. 2016;14: 20–32. doi:10.1038/nrmicro3552

52.	Croom J, Chichlowski M, Froetschel M, McBride BW, Qui R, Koci MD. The effects of direct-fed microbial, primalac, or salinomycin supplementation on intestinal lactate isomers and cecal volatile fatty acid concentrations in broilers. Int J Poult Sci. 2009;8: 128–132. doi:10.3923/IJPS.2009.128.132

56.	Rada V, Marounek M. Effect of monensin on the crop microflora of broiler chickens. Ann Zootech. 1996;45: 283–288. doi:10.1051/animres:19960308

67.	Elwinger K, Berndtson E, Engström B, Fossum O, Waldenstedt L. Effect of antibiotic growth promoters and anticoccidials on growth of *Clostridium perfringens* in the caeca and on performance of broiler chickens. Acta Vet Scand. 1998;39: 433–441. doi:10.1186/BF03547769

- The references were changed for the reference list numbers 40 and 45.

40.	Karunaratne ND, Classen HL, Ames NP, Bedford MR, Newkirk RW. Effects of hulless barley and exogenous beta-glucanase levels on ileal digesta soluble beta-glucan molecular weight, digestive tract characteristics, and performance of broiler chickens. Poult Sci. 2021;100: 100967. doi:10.1016/j.psj.2020.12.064

45.	Karunaratne ND, Newkirk RW, Van Kessel AG, Bedford MR, Classen HL. Hulless barley and beta-glucanase levels in the diet affect the performance of coccidiosis-challenged broiler chickens in an age-dependent manner. Poult Sci. 2021;100: 776–787. doi:10.1016/j.psj.2020.10.036

- The reference number 69 was removed, and the text was updated.

69.	Karunaratne ND, Bedford MR, Newkirk RW, Classen HL. Graded levels of hulless barley and β-glucanase affect the performance of broiler chickens vaccinated for coccidiosis in an age dependent manner. Poult Sci. 2017b;96 (E-suppl. 1): 50.

**Reviewer #1**

| Line | Comment |
| --- | --- |
| L26-48 | Abstract; It may be easier for the reader to understand the content if the abstract is divided into items as shown below.  The abstract was divided into items as requested (L 27-49). |
| L30, L47, L350, Table9, L519, Table20, L665, L687, L688, L703, L707, L710, L711, L714, and L728 | "Performance," which is often used throughout, is an ambiguous expression that hinders the content's objective understanding. They should specifically indicate "bodyweight," "feed intake," etc., respectively.  Revised using the terms ‘body weight gain’, ‘feed intake’ and ‘feed to gain ratio/feed efficiency’ (L 353, 523, 669, 691, 708, 712, 715, 716, 724, 733-734).  Changed as ‘growth performance’ because the length of the abstract increase if use all the terms (L 30, 48). |
| L104-114 | The content of the individual experiments and the results of conducting them are objectively presented in the main contents of the present study. L104-114, which is an inference of subjective and concreted experimental results, should be deleted.  This statement was included in the objective and hypothesis section to clearly emphasis the reason for conducting two experiments in the current study as requested by Reviewer #2 in the previous reviewing process (L 104-115). No changes were made. |
| L305, Table3, L391 | “Viscosity” should be replaced with “The viscosity of ileal supernatant” indicated by the material and method. Please unify the terms.  Revised as “The viscosity of ileal supernatant’ (L 306, Table 3, 394). |
| L478 | The weight of the contents of the small intestine is not the result of morphological observation of the digestive tract, but the result of "measurement of the contents of the small intestine". Heading titles must be separated.  A separate heading title was added for the content weights as “measurements of the contents of the digestive tract’ (L 339). |
| L483 | As with the L478, the headings need to be separated.  A separate heading title was added for the content weights as “measurements of the contents of the digestive tract, and digestive organ morphology’ (L 491-492). The table numbers 17 and 18 were switched because of the changes of the content weight paragraphs (L 483, 494). |
| L467: L467-502 | Alternatively, it may be appropriate to say, for example, "digestive organ morphology and weight of contents" instead of "digestive tract morphology."  The digestive tract morphology and content weights sections were separated, and separate heading titles were added as mentioned in the previous two comments (L 339, 491-492). |
| L604-605 | I think "ileal viscosity" is probably not an accurate expression. Is the ileum flexible? Is it the viscosity of the gastrointestinal contents (ileal supernatant)?  Revised as ‘viscosity of the ileal supernatant’ (L 609-610). |
| L691-692 | What does growth performance mean? What is the positive influence?  Revised as ‘increased body weight gain and feed conversion’ (L 696). |
| L687, L714 | I don't think "performance variables" and "production cycle" are appropriate words to use in this context. Specific items used as performance indicators should be shown individually. Also, the term production cycle used here seems to refer to the rearing period, inferring from the context.  The terms ‘body weight gain, feed intake and feed efficiency’ was used instead of ‘performance variables’ (L 691, 719-720).  The term ‘production cycle’ was removed and the sentence was revised (L 693). |
